# Tris(1,10-phenanthroline)iron(II) μ-oxido-bis­[trichloridoferrate(III)]

**DOI:** 10.1107/S1600536811031783

**Published:** 2011-08-11

**Authors:** Chun Ling, Li Song, Xinping Wang

**Affiliations:** a Department of Chemistry, Key Laboratory of Advanced Textile Materials and Manufacturing Technology of the Education Ministry, Zhejiang Sci-Tech University, Hangzhou 310018, People’s Republic of China

## Abstract

In the title salt, [Fe(C_12_H_8_N_2_)_3_][Fe_2_Cl_6_O], the ionic components are linked into a two-dimensional supra­molecular layer by two pairs of C—H⋯Cl hydrogen bonds and π–π stacking inter­actions [centroid–centroid distances = 3.655 (4) and 3.498 (3) Å]. The salt is characterized as a mixed-valent Fe^II^–Fe^III^ compound, in which an Fe^II^ atom is coordinated by three phen ligands, forming a six-coordinated cationic entity and the anionic part is formed by two Fe^III^ atoms in tetra­hedral coordination environments constructed by three chloride ions and one bridging oxide ligand. Intra­molecular C—H⋯N hydrogen bonds are observed.

## Related literature

For related compounds containing the [Cl_3_FeOFeCl_3_]^2−^ anion, see: Yan *et al.* (2000 ▶); Li *et al.* (2008 ▶); Haselhorst *et al.* (1993 ▶); Drew *et al.* (1978 ▶); Ondrejkovicová *et al.* (1998 ▶); James *et al.* (1997 ▶); Köhn *et al.* (1997 ▶); Bullen *et al.* (1986 ▶). For polynuclear iron(II/III) clusters, see: Pierre *et al.* (1996 ▶); Proul-Curry & Chasteen (1995 ▶). For the use of iron(III) complexes containing an Fe—O—Fe linkage as models for non-heme metalloproteins, see: Kurtz (1990 ▶); Gorun & Lippard (1991 ▶); Davydov *et al.* (1997 ▶); Ito *et al.* (1996 ▶); Mauerer *et al.* (1993 ▶); Menage *et al.* (1993 ▶); Okuno *et al.* (1997 ▶). For their use as models in studies of intra­molecular anti­ferromagnetic spin exchange coupling between high-spin ferric ions in material science, see: Kurtz (1990 ▶); Gatteschi *et al.* (2000 ▶); Haselhorst *et al.* (1993 ▶). For π–π stacking inter­actions between two phen ligands, see: Chandrasekhar *et al.* (2006 ▶). 
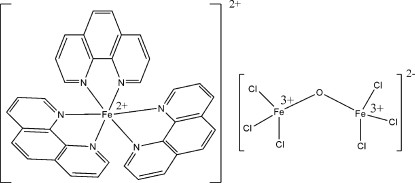


## Experimental

### 

#### Crystal data

[Fe(C_12_H_8_N_2_)_3_][Fe_2_Cl_6_O]
*M* *_r_* = 936.86
Triclinic, 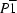

*a* = 11.422 (2) Å
*b* = 13.357 (3) Å
*c* = 14.045 (3) Å
α = 77.61 (3)°
β = 89.16 (3)°
γ = 65.99 (3)°
*V* = 1905.3 (7) Å^3^
*Z* = 2
Mo *K*α radiation
μ = 1.59 mm^−1^
*T* = 293 K
0.38 × 0.20 × 0.12 mm

#### Data collection

Rigaku R-AXIS RAPID diffractometer
Absorption correction: multi-scan (*ABSCOR*; Higashi, 1995 ▶) *T*_min_ = 0.584, *T*_max_ = 0.832
18867 measured reflections
8629 independent reflections
5284 reflections with *I* > 2σ(*I*)
*R*_int_ = 0.038

#### Refinement

*R*[*F* ^2^ > 2σ(*F* ^2^)] = 0.050
*wR*(*F* ^2^) = 0.172
*S* = 1.14
8629 reflections
469 parameters
H-atom parameters constrained
Δρ_max_ = 1.02 e Å^−3^
Δρ_min_ = −1.14 e Å^−3^

### 

Data collection: *PROCESS-AUTO* (Rigaku, 1998 ▶); cell refinement: *PROCESS-AUTO*; data reduction: *CrystalStructure* (Rigaku/MSC, 2004 ▶); program(s) used to solve structure: *SHELXS97* (Sheldrick, 2008 ▶); program(s) used to refine structure: *SHELXL97* (Sheldrick, 2008 ▶); molecular graphics: *SHELXTL* (Sheldrick, 2008 ▶); software used to prepare material for publication: *SHELXTL*.

## Supplementary Material

Crystal structure: contains datablock(s) I, global. DOI: 10.1107/S1600536811031783/bg2417sup1.cif

Structure factors: contains datablock(s) I. DOI: 10.1107/S1600536811031783/bg2417Isup2.hkl

Additional supplementary materials:  crystallographic information; 3D view; checkCIF report

## Figures and Tables

**Table 1:** Hydrogen-bond geometry (Å, °)

*D*—H⋯*A*	*D*—H	H⋯*A*	*D*⋯*A*	*D*—H⋯*A*
C1—H1⋯Cl6^i^	0.93	2.80	3.416 (7)	125
C11—H11⋯Cl2	0.93	2.82	3.740 (7)	172
C12—H12⋯N4	0.93	2.55	3.038 (7)	113
C25—H25⋯N3	0.93	2.62	3.098 (7)	113
C36—H36⋯N2	0.93	2.60	3.084 (8)	113

Symmetry code: (i) 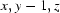.

**Fig. 1.**
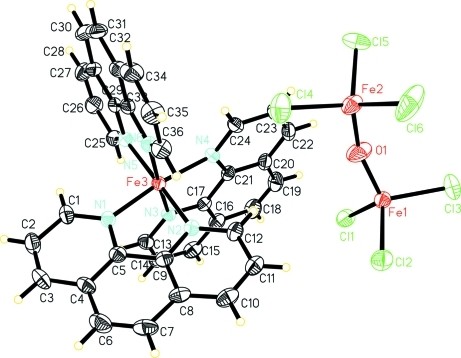
Structure and labeling of the title compound, with displacement ellipsoids drawn at the 30% probability level and H atoms shown as small spheres of arbitrary radii.

**Fig. 2.**
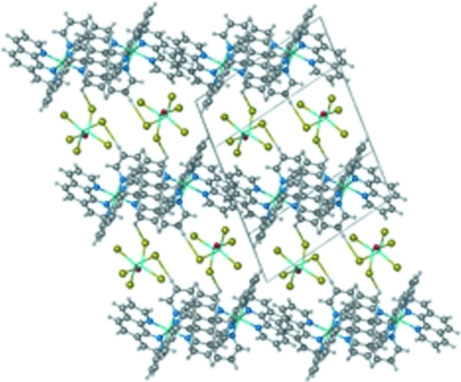
The supramolecular organic-inorganic hybrid layer constructed by π-π stacking interactions and hydrogen bonds.

**Fig. 3.**
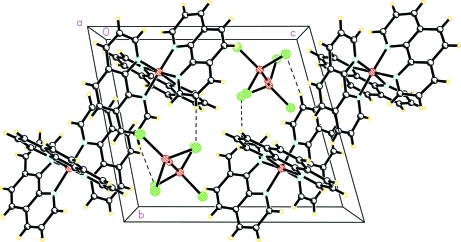
The packing diagram viewed along the a-direction.

## supplementary crystallographic information

### Comment

Recently polynuclear iron(II/III) clusters have received considerable attention in inorganic chemistry and material science (Proul-Curry *et al.*, 1995; Pierre *et al.*, 1996). In particular, iron(III) complexes containing Fe—O—Fe linkage have been one of the more celebrated objects for research and exploiture. In bioioganic chemistry, they are simple and useful models for non-heme metalloproteins containing dinuclear iron units in their active site, such as the methane monooxygenase, hemerythrin, etc (Kurtz *et al.*, 1990; Gorun *et al.*, 1991; Davydov *et al.*, 1997). In material science, they have also been considered as useful models in studies of intramolecular antiferromagnetic spin exchange coupling between high-spin ferric ions (Kurtz *et al.*, 1990; Haselhorst *et al.*, 1993; Gatteschi *et al.*, 2000). Previously, many efforts have been contributed to these researches, especially to the models for non-heme metalloproteins (Davydov *et al.*, 1997; Mauerer *et al.*, 1993; Ito *et al.*, 1996; Okuno *et al.*, 1997; Menage *et al.*, 1993). Here, we report a ionic compound, [Fe(phen)_3_][Cl_3_FeOFeCl_3_] (I), composed of a dinuclear Fe^III^ cluster anion, [Cl_3_FeOFeCl_3_]^2-^, and a coordinated cation containing Fe^II^, [Fe(phen)_3_]^2+^.

The Fe^II^ centre is coordinated in octahedral geometry by three phen ligands to form a coordination cation. In this FeN_6_ octahedron, Fe—N bond lengths range from 1.972 (4) Å to 1.985 (4) Å and are similar to those reported in the literature (Yan *et al.*, 2000; Li *et al.*, 2008). In the anionic group two Fe^III^ cations locate in similar tetrahedral environments constructed by three Cl_-_ and one µ_2_-bridged O^2-^ ligand. Fe—Cl bond lengths range from 2.206 (2) Å to 2.247 (2) Å and are similar to those in the literature (Haselhorst *et al.*, 1993; Drew *et al.*, 1978; Ondrejkovicová *et al.*, 1998; James *et al.*, 1997; Köhn *et al.*, 1997; Bullen *et al.*, 1986). These two FeOCl_3_ tetrahedra are fused through the µ_2_-bridged O^2-^ ligand (Fe1—O1 = 1.747 (4) Å, Fe2—O1 = 1.753 (4) Å ) to give out a dinuclear cluster.

In the crystal structure offset face-to face aromatic π-π stacking interactions and hydrogen bonds lead to the formation of a two-dimensional supramolecular layer. Firstly, along the [1 - 1 1] direction, all adjacent cation of [Fe(phen)_3_]^2+^ are joined to each other by virtue of π–π stacking interactions between two phen ligands to form a one-dimensional supramolecular chain (Chandrasekhar *et al.*, 2006). Two pairs of phen skeletons are arranged in a parallel fashion, ring 1 (C4—C9) of one cation stacks with ring 2 (C4—C9)^i ^[(i): 2 - *x*, -*y*, 1 - *z*] of a neighbouring cation with an interplanar distance of 3.487 (9) Å, and ring 3 (N4/C20—C24) of one cation stacks with ring 4 (N4/C20—C24)^ii^ [(ii) 1 - *x*, 1 - *y*, -*z*] of a neighbouring cation with an interplanar distance of 3.250 (6) Å. Adjacent chains, in turn, are fused together by the [Cl_3_FeOFeCl_3_]^2-^inorganic anion through two pairs of (C—H···Cl) hydrogen bonding interactions between cations and anions (Table 1). As a result, the supramolecular chains interconnect to form a two-dimensional supramolecular layer.

### Experimental

The title compound (I) was synthesized by solvothermal reaction of FeCl_2_ tetrahydrate (20 mg, 0.1 mmol), Et_4_NBr (21 mg, 0.1 mmol), α-Ketoglutaric acid (15 mg, 0.1 mmol) and 1,10-phenanthroline monohydrate (20 mg, 0.1 mmol) in 6 mL e thanol and 0.5 ml water containing NaOH (4 mg, 0.1 mmol). The mixture was heated to 373 K at a rate of 20 K/h, and kept at this temperature for 1 day and then cooled to room temperature at a rate of 2 K/h. Dark red crystals of (I) were obtained. Anal. Calc. for C36H24Cl6Fe3N6O (%): C, 46.15; H, 2.58; N, 8.97; O, 1.71. Found: C, 42.58; H, 2.73; N, 8.36;O, 1.97. Crystals of (I) suitable for single-crystal X-ray diffraction were selected directly from the sample as prepared.

### Refinement

All hydrogen atoms were added at calculated positions and refined using a riding model (C-H: 0.93Å, U(H): 1.2 × U_eq_(C).

### Crystal data

**Table:**

[Fe(C_12_H_8_N_2_)_3_][Fe_2_Cl_6_O]	*V* = 1905.3 (7) Å^3^
*M**_r_* = 936.86	*Z* = 2
Triclinic, *P*1	*F*(000) = 940
Hall symbol: -P 1	*D*_x_ = 1.633 Mg m^−^^3^
*a* = 11.422 (2) Å	Mo *K*α radiation, λ = 0.71075 Å
*b* = 13.357 (3) Å	θ = 3.1–27.4°
*c* = 14.045 (3) Å	µ = 1.59 mm^−^^1^
α = 77.61 (3)°	*T* = 293 K
β = 89.16 (3)°	Chunk, dark red
γ = 65.99 (3)°	0.38 × 0.20 × 0.12 mm

### Data collection

**Table:**

Rigaku R-AXIS RAPID diffractometer	8629 independent reflections
Radiation source: fine-focus sealed tube	5284 reflections with *I* > 2σ(*I*)
graphite	*R*_int_ = 0.038
Detector resolution: 14.6306 pixels mm^-1^	θ_max_ = 27.4°, θ_min_ = 3.1°
CCD_Profile_fitting scans	*h* = −14→14
Absorption correction: multi-scan (*ABSCOR*; Higashi, 1995)	*k* = −16→17
*T*_min_ = 0.584, *T*_max_ = 0.832	*l* = −18→18
18867 measured reflections	

### Refinement

**Table:**

Refinement on *F*^2^	Primary atom site location: structure-invariant direct methods
Least-squares matrix: full	Secondary atom site location: difference Fourier map
*R*[*F*^2^ > 2σ(*F*^2^)] = 0.050	Hydrogen site location: inferred from neighbouring sites
*wR*(*F*^2^) = 0.172	H-atom parameters constrained
*S* = 1.14	*w* = 1/[σ^2^(*F*_o_^2^) + (0.0663*P*)^2^ + 2.5229*P*] where *P* = (*F*_o_^2^ + 2*F*_c_^2^)/3
8629 reflections	(Δ/σ)_max_ < 0.001
469 parameters	Δρ_max_ = 1.02 e Å^−^^3^
0 restraints	Δρ_min_ = −1.14 e Å^−^^3^

### Special details

**Table:**

Geometry. All e.s.d.'s (except the e.s.d. in the dihedral angle between two l.s. planes) are estimated using the full covariance matrix. The cell e.s.d.'s are taken into account individually in the estimation of e.s.d.'s in distances, angles and torsion angles; correlations between e.s.d.'s in cell parameters are only used when they are defined by crystal symmetry. An approximate (isotropic) treatment of cell e.s.d.'s is used for estimating e.s.d.'s involving l.s. planes.
Refinement. Refinement of *F*^2^ against ALL reflections. The weighted *R*-factor *wR* and goodness of fit *S* are based on *F*^2^, conventional *R*-factors *R* are based on *F*, with *F* set to zero for negative *F*^2^. The threshold expression of *F*^2^ > σ(*F*^2^) is used only for calculating *R*-factors(gt) *etc*. and is not relevant to the choice of reflections for refinement. *R*-factors based on *F*^2^ are statistically about twice as large as those based on *F*, and *R*- factors based on ALL data will be even larger.

### Fractional atomic coordinates and isotropic or equivalent isotropic displacement parameters (Å^2^)

**Table:**

	*x*	*y*	*z*	*U*_iso_*/*U*_eq_	
Fe1	0.77271 (7)	0.70380 (6)	0.21753 (6)	0.0453 (2)	
Fe2	0.45222 (7)	0.78460 (8)	0.25534 (7)	0.0592 (2)	
Fe3	0.63944 (6)	0.23329 (5)	0.26545 (5)	0.03354 (17)	
Cl1	0.87079 (12)	0.57349 (12)	0.13308 (12)	0.0588 (4)	
Cl2	0.88071 (16)	0.64218 (14)	0.36574 (11)	0.0678 (4)	
Cl3	0.78594 (18)	0.86418 (12)	0.14353 (12)	0.0726 (5)	
Cl4	0.4048 (2)	0.64893 (16)	0.34770 (13)	0.0815 (5)	
Cl5	0.31464 (15)	0.86581 (16)	0.12050 (13)	0.0815 (5)	
Cl6	0.4309 (2)	0.9143 (3)	0.3368 (2)	0.1410 (12)	
O1	0.6116 (4)	0.7255 (4)	0.2250 (4)	0.0808 (14)	
N1	0.6923 (4)	0.0922 (3)	0.3668 (3)	0.0388 (8)	
N2	0.7466 (4)	0.2671 (3)	0.3528 (3)	0.0376 (8)	
N3	0.7857 (3)	0.1709 (3)	0.1870 (3)	0.0350 (8)	
N4	0.6082 (4)	0.3749 (3)	0.1681 (3)	0.0372 (8)	
N5	0.5217 (4)	0.2003 (3)	0.1873 (3)	0.0388 (8)	
N6	0.4822 (3)	0.3024 (3)	0.3321 (3)	0.0390 (9)	
C1	0.6653 (5)	0.0032 (4)	0.3706 (4)	0.0504 (12)	
H1	0.6160	0.0030	0.3186	0.060*	
C2	0.7092 (6)	−0.0902 (5)	0.4507 (4)	0.0620 (15)	
H2	0.6884	−0.1508	0.4513	0.074*	
C3	0.7815 (6)	−0.0918 (5)	0.5265 (4)	0.0633 (15)	
H3	0.8112	−0.1536	0.5793	0.076*	
C4	0.8118 (5)	0.0006 (4)	0.5251 (4)	0.0497 (12)	
C5	0.7658 (4)	0.0887 (4)	0.4445 (4)	0.0420 (11)	
C6	0.8841 (6)	0.0092 (6)	0.6028 (4)	0.0688 (17)	
H6	0.9149	−0.0494	0.6582	0.083*	
C7	0.9084 (6)	0.1003 (6)	0.5973 (4)	0.0676 (17)	
H7	0.9538	0.1042	0.6497	0.081*	
C8	0.8658 (5)	0.1921 (5)	0.5122 (4)	0.0504 (12)	
C9	0.7944 (4)	0.1850 (4)	0.4368 (4)	0.0416 (11)	
C10	0.8893 (5)	0.2893 (5)	0.5004 (4)	0.0602 (15)	
H10	0.9346	0.2985	0.5499	0.072*	
C11	0.8450 (5)	0.3699 (5)	0.4156 (4)	0.0564 (14)	
H11	0.8628	0.4333	0.4058	0.068*	
C12	0.7732 (5)	0.3570 (5)	0.3440 (4)	0.0494 (12)	
H12	0.7422	0.4136	0.2873	0.059*	
C13	0.8717 (4)	0.0659 (4)	0.1962 (4)	0.0451 (11)	
H13	0.8637	0.0097	0.2444	0.054*	
C14	0.9742 (5)	0.0355 (5)	0.1367 (4)	0.0533 (13)	
H14	1.0332	−0.0392	0.1462	0.064*	
C15	0.9870 (5)	0.1160 (5)	0.0647 (4)	0.0562 (14)	
H15	1.0552	0.0968	0.0252	0.067*	
C16	0.8967 (5)	0.2280 (5)	0.0505 (4)	0.0488 (12)	
C17	0.7975 (4)	0.2507 (4)	0.1139 (3)	0.0363 (10)	
C18	0.8991 (6)	0.3197 (6)	−0.0222 (4)	0.0624 (16)	
H18	0.9632	0.3063	−0.0655	0.075*	
C19	0.8106 (6)	0.4249 (5)	−0.0291 (4)	0.0588 (15)	
H19	0.8164	0.4832	−0.0759	0.071*	
C20	0.7072 (5)	0.4504 (4)	0.0336 (4)	0.0463 (12)	
C21	0.7017 (4)	0.3619 (4)	0.1050 (3)	0.0379 (10)	
C22	0.6122 (6)	0.5585 (4)	0.0306 (4)	0.0522 (13)	
H22	0.6130	0.6204	−0.0144	0.063*	
C23	0.5188 (5)	0.5719 (4)	0.0942 (4)	0.0503 (12)	
H23	0.4551	0.6432	0.0928	0.060*	
C24	0.5192 (5)	0.4778 (4)	0.1619 (4)	0.0428 (11)	
H24	0.4540	0.4882	0.2043	0.051*	
C25	0.5450 (5)	0.1474 (4)	0.1150 (4)	0.0455 (11)	
H25	0.6290	0.1156	0.0977	0.055*	
C26	0.4467 (6)	0.1377 (5)	0.0631 (4)	0.0591 (14)	
H26	0.4662	0.1009	0.0119	0.071*	
C27	0.3247 (6)	0.1815 (5)	0.0876 (5)	0.0640 (16)	
H27	0.2602	0.1735	0.0545	0.077*	
C28	0.2949 (5)	0.2394 (4)	0.1632 (4)	0.0498 (12)	
C29	0.3974 (4)	0.2457 (4)	0.2119 (4)	0.0397 (10)	
C30	0.1686 (5)	0.2942 (6)	0.1940 (5)	0.0660 (17)	
H30	0.0989	0.2925	0.1622	0.079*	
C31	0.1479 (5)	0.3481 (5)	0.2676 (5)	0.0646 (17)	
H31	0.0644	0.3830	0.2849	0.077*	
C32	0.2513 (5)	0.3525 (4)	0.3196 (4)	0.0506 (13)	
C33	0.3761 (4)	0.3017 (4)	0.2895 (4)	0.0408 (10)	
C34	0.2381 (5)	0.4033 (5)	0.3984 (5)	0.0640 (16)	
H34	0.1575	0.4389	0.4201	0.077*	
C35	0.3444 (6)	0.4002 (5)	0.4429 (5)	0.0634 (16)	
H35	0.3369	0.4314	0.4971	0.076*	
C36	0.4660 (5)	0.3501 (5)	0.4081 (4)	0.0517 (13)	
H36	0.5371	0.3505	0.4391	0.062*	

### Atomic displacement parameters (Å^2^)

**Table:**

	*U*^11^	*U*^22^	*U*^33^	*U*^12^	*U*^13^	*U*^23^
Fe1	0.0378 (4)	0.0458 (4)	0.0546 (5)	−0.0166 (3)	0.0088 (3)	−0.0176 (3)
Fe2	0.0421 (4)	0.0729 (6)	0.0750 (6)	−0.0270 (4)	0.0207 (4)	−0.0364 (5)
Fe3	0.0321 (3)	0.0315 (3)	0.0363 (4)	−0.0117 (3)	0.0047 (3)	−0.0094 (3)
Cl1	0.0447 (7)	0.0568 (8)	0.0797 (10)	−0.0154 (6)	0.0089 (7)	−0.0369 (7)
Cl2	0.0802 (10)	0.0777 (10)	0.0498 (8)	−0.0375 (9)	−0.0072 (7)	−0.0122 (7)
Cl3	0.1030 (12)	0.0447 (8)	0.0744 (10)	−0.0344 (9)	0.0205 (9)	−0.0150 (7)
Cl4	0.1150 (14)	0.0870 (12)	0.0655 (10)	−0.0621 (12)	0.0340 (10)	−0.0241 (9)
Cl5	0.0482 (8)	0.0802 (11)	0.0802 (11)	0.0078 (8)	−0.0025 (8)	−0.0149 (9)
Cl6	0.1111 (16)	0.183 (3)	0.226 (3)	−0.1023 (18)	0.0912 (19)	−0.164 (3)
O1	0.041 (2)	0.104 (4)	0.107 (4)	−0.027 (2)	0.020 (2)	−0.049 (3)
N1	0.040 (2)	0.037 (2)	0.040 (2)	−0.0161 (18)	0.0058 (17)	−0.0100 (17)
N2	0.0375 (19)	0.038 (2)	0.038 (2)	−0.0161 (17)	0.0071 (17)	−0.0113 (17)
N3	0.0319 (18)	0.0342 (19)	0.038 (2)	−0.0109 (16)	0.0040 (16)	−0.0126 (16)
N4	0.0381 (19)	0.032 (2)	0.037 (2)	−0.0106 (17)	0.0030 (16)	−0.0069 (16)
N5	0.039 (2)	0.035 (2)	0.041 (2)	−0.0148 (18)	0.0003 (17)	−0.0065 (17)
N6	0.0362 (19)	0.034 (2)	0.046 (2)	−0.0123 (17)	0.0098 (17)	−0.0128 (17)
C1	0.059 (3)	0.037 (3)	0.056 (3)	−0.023 (3)	0.008 (3)	−0.007 (2)
C2	0.079 (4)	0.046 (3)	0.063 (4)	−0.033 (3)	0.004 (3)	−0.003 (3)
C3	0.075 (4)	0.042 (3)	0.054 (3)	−0.015 (3)	−0.002 (3)	0.008 (3)
C4	0.052 (3)	0.047 (3)	0.035 (3)	−0.012 (3)	−0.001 (2)	0.003 (2)
C5	0.035 (2)	0.044 (3)	0.044 (3)	−0.012 (2)	0.008 (2)	−0.011 (2)
C6	0.072 (4)	0.068 (4)	0.048 (3)	−0.021 (3)	−0.008 (3)	0.007 (3)
C7	0.059 (3)	0.086 (5)	0.047 (3)	−0.022 (3)	−0.018 (3)	−0.006 (3)
C8	0.039 (3)	0.067 (4)	0.044 (3)	−0.019 (3)	−0.004 (2)	−0.019 (3)
C9	0.034 (2)	0.049 (3)	0.042 (3)	−0.015 (2)	0.010 (2)	−0.015 (2)
C10	0.055 (3)	0.081 (4)	0.058 (4)	−0.033 (3)	−0.001 (3)	−0.032 (3)
C11	0.055 (3)	0.063 (4)	0.063 (4)	−0.033 (3)	0.003 (3)	−0.022 (3)
C12	0.056 (3)	0.051 (3)	0.053 (3)	−0.030 (3)	0.006 (2)	−0.019 (2)
C13	0.040 (2)	0.040 (3)	0.050 (3)	−0.011 (2)	0.006 (2)	−0.014 (2)
C14	0.040 (3)	0.052 (3)	0.062 (3)	−0.009 (2)	0.007 (2)	−0.023 (3)
C15	0.039 (3)	0.074 (4)	0.063 (4)	−0.022 (3)	0.021 (3)	−0.035 (3)
C16	0.043 (3)	0.060 (3)	0.050 (3)	−0.025 (3)	0.012 (2)	−0.019 (3)
C17	0.034 (2)	0.042 (3)	0.037 (2)	−0.018 (2)	0.0058 (19)	−0.014 (2)
C18	0.067 (4)	0.077 (4)	0.056 (4)	−0.042 (4)	0.025 (3)	−0.018 (3)
C19	0.075 (4)	0.065 (4)	0.047 (3)	−0.044 (3)	0.017 (3)	−0.007 (3)
C20	0.055 (3)	0.048 (3)	0.043 (3)	−0.031 (3)	−0.001 (2)	−0.004 (2)
C21	0.041 (2)	0.042 (3)	0.036 (2)	−0.022 (2)	0.002 (2)	−0.010 (2)
C22	0.072 (4)	0.045 (3)	0.047 (3)	−0.035 (3)	−0.006 (3)	−0.001 (2)
C23	0.060 (3)	0.030 (2)	0.058 (3)	−0.014 (2)	−0.007 (3)	−0.011 (2)
C24	0.043 (3)	0.035 (3)	0.044 (3)	−0.010 (2)	−0.002 (2)	−0.007 (2)
C25	0.050 (3)	0.041 (3)	0.048 (3)	−0.016 (2)	−0.002 (2)	−0.020 (2)
C26	0.067 (4)	0.057 (3)	0.060 (4)	−0.027 (3)	−0.008 (3)	−0.020 (3)
C27	0.065 (4)	0.064 (4)	0.073 (4)	−0.039 (3)	−0.008 (3)	−0.011 (3)
C28	0.039 (3)	0.050 (3)	0.055 (3)	−0.020 (2)	−0.009 (2)	0.005 (2)
C29	0.036 (2)	0.034 (2)	0.046 (3)	−0.016 (2)	0.003 (2)	0.000 (2)
C30	0.041 (3)	0.084 (4)	0.068 (4)	−0.032 (3)	−0.006 (3)	0.006 (3)
C31	0.031 (3)	0.078 (4)	0.064 (4)	−0.014 (3)	0.003 (3)	0.008 (3)
C32	0.037 (2)	0.049 (3)	0.052 (3)	−0.011 (2)	0.010 (2)	0.003 (2)
C33	0.037 (2)	0.035 (2)	0.046 (3)	−0.013 (2)	0.008 (2)	−0.003 (2)
C34	0.045 (3)	0.065 (4)	0.067 (4)	−0.007 (3)	0.024 (3)	−0.016 (3)
C35	0.063 (4)	0.066 (4)	0.059 (4)	−0.018 (3)	0.028 (3)	−0.028 (3)
C36	0.053 (3)	0.054 (3)	0.049 (3)	−0.019 (3)	0.013 (2)	−0.021 (3)

### Geometric parameters (Å, °)

**Table:**

Fe1—O1	1.747 (4)	C11—C12	1.388 (7)
Fe1—Cl1	2.2251 (16)	C11—H11	0.9300
Fe1—Cl3	2.2350 (17)	C12—H12	0.9300
Fe1—Cl2	2.2463 (19)	C13—C14	1.402 (7)
Fe2—O1	1.753 (4)	C13—H13	0.9300
Fe2—Cl6	2.206 (2)	C14—C15	1.363 (8)
Fe2—Cl4	2.2424 (19)	C14—H14	0.9300
Fe2—Cl5	2.247 (2)	C15—C16	1.401 (8)
Fe3—N1	1.972 (4)	C15—H15	0.9300
Fe3—N3	1.976 (4)	C16—C17	1.405 (6)
Fe3—N6	1.981 (4)	C16—C18	1.427 (8)
Fe3—N4	1.983 (4)	C17—C21	1.421 (6)
Fe3—N2	1.985 (4)	C18—C19	1.339 (8)
Fe3—N5	1.985 (4)	C18—H18	0.9300
N1—C1	1.335 (6)	C19—C20	1.434 (7)
N1—C5	1.367 (6)	C19—H19	0.9300
N2—C12	1.334 (6)	C20—C21	1.400 (6)
N2—C9	1.367 (6)	C20—C22	1.401 (8)
N3—C13	1.324 (6)	C22—C23	1.361 (7)
N3—C17	1.361 (6)	C22—H22	0.9300
N4—C24	1.320 (6)	C23—C24	1.403 (7)
N4—C21	1.357 (6)	C23—H23	0.9300
N5—C25	1.322 (6)	C24—H24	0.9300
N5—C29	1.370 (6)	C25—C26	1.412 (7)
N6—C36	1.331 (6)	C25—H25	0.9300
N6—C33	1.363 (6)	C26—C27	1.346 (8)
C1—C2	1.407 (8)	C26—H26	0.9300
C1—H1	0.9300	C27—C28	1.403 (8)
C2—C3	1.348 (8)	C27—H27	0.9300
C2—H2	0.9300	C28—C29	1.405 (7)
C3—C4	1.407 (8)	C28—C30	1.437 (8)
C3—H3	0.9300	C29—C33	1.414 (7)
C4—C5	1.373 (7)	C30—C31	1.347 (9)
C4—C6	1.430 (8)	C30—H30	0.9300
C5—C9	1.433 (7)	C31—C32	1.427 (8)
C6—C7	1.339 (9)	C31—H31	0.9300
C6—H6	0.9300	C32—C34	1.393 (8)
C7—C8	1.439 (8)	C32—C33	1.410 (6)
C7—H7	0.9300	C34—C35	1.354 (9)
C8—C9	1.389 (7)	C34—H34	0.9300
C8—C10	1.405 (8)	C35—C36	1.407 (7)
C10—C11	1.363 (8)	C35—H35	0.9300
C10—H10	0.9300	C36—H36	0.9300
			
O1—Fe1—Cl1	110.07 (16)	C10—C11—H11	120.2
O1—Fe1—Cl3	110.06 (18)	C12—C11—H11	120.2
Cl1—Fe1—Cl3	109.18 (7)	N2—C12—C11	123.1 (5)
O1—Fe1—Cl2	112.02 (18)	N2—C12—H12	118.5
Cl1—Fe1—Cl2	106.97 (7)	C11—C12—H12	118.5
Cl3—Fe1—Cl2	108.45 (7)	N3—C13—C14	123.0 (5)
O1—Fe2—Cl6	108.97 (16)	N3—C13—H13	118.5
O1—Fe2—Cl4	109.22 (18)	C14—C13—H13	118.5
Cl6—Fe2—Cl4	110.12 (10)	C15—C14—C13	119.6 (5)
O1—Fe2—Cl5	111.14 (18)	C15—C14—H14	120.2
Cl6—Fe2—Cl5	108.54 (12)	C13—C14—H14	120.2
Cl4—Fe2—Cl5	108.85 (8)	C14—C15—C16	119.5 (4)
N1—Fe3—N3	93.83 (16)	C14—C15—H15	120.2
N1—Fe3—N6	90.17 (16)	C16—C15—H15	120.2
N3—Fe3—N6	174.48 (16)	C15—C16—C17	116.9 (5)
N1—Fe3—N4	172.55 (16)	C15—C16—C18	124.9 (5)
N3—Fe3—N4	82.51 (15)	C17—C16—C18	118.1 (5)
N6—Fe3—N4	93.94 (16)	N3—C17—C16	123.7 (4)
N1—Fe3—N2	82.86 (16)	N3—C17—C21	115.4 (4)
N3—Fe3—N2	91.56 (15)	C16—C17—C21	120.8 (4)
N6—Fe3—N2	92.71 (15)	C19—C18—C16	121.0 (5)
N4—Fe3—N2	90.73 (16)	C19—C18—H18	119.5
N1—Fe3—N5	95.14 (16)	C16—C18—H18	119.5
N3—Fe3—N5	93.20 (15)	C18—C19—C20	122.1 (5)
N6—Fe3—N5	82.65 (16)	C18—C19—H19	119.0
N4—Fe3—N5	91.54 (16)	C20—C19—H19	119.0
N2—Fe3—N5	174.96 (15)	C21—C20—C22	116.9 (5)
Fe1—O1—Fe2	158.4 (3)	C21—C20—C19	118.1 (5)
C1—N1—C5	117.0 (4)	C22—C20—C19	124.9 (5)
C1—N1—Fe3	129.7 (4)	N4—C21—C20	123.8 (4)
C5—N1—Fe3	113.3 (3)	N4—C21—C17	116.5 (4)
C12—N2—C9	117.1 (4)	C20—C21—C17	119.8 (4)
C12—N2—Fe3	130.6 (4)	C23—C22—C20	119.4 (5)
C9—N2—Fe3	112.3 (3)	C23—C22—H22	120.3
C13—N3—C17	117.1 (4)	C20—C22—H22	120.3
C13—N3—Fe3	129.8 (3)	C22—C23—C24	119.7 (5)
C17—N3—Fe3	113.0 (3)	C22—C23—H23	120.2
C24—N4—C21	117.4 (4)	C24—C23—H23	120.2
C24—N4—Fe3	129.9 (3)	N4—C24—C23	122.7 (5)
C21—N4—Fe3	112.3 (3)	N4—C24—H24	118.6
C25—N5—C29	117.9 (4)	C23—C24—H24	118.6
C25—N5—Fe3	129.9 (3)	N5—C25—C26	122.2 (5)
C29—N5—Fe3	112.1 (3)	N5—C25—H25	118.9
C36—N6—C33	117.4 (4)	C26—C25—H25	118.9
C36—N6—Fe3	129.9 (3)	C27—C26—C25	120.1 (5)
C33—N6—Fe3	112.6 (3)	C27—C26—H26	119.9
N1—C1—C2	122.0 (5)	C25—C26—H26	119.9
N1—C1—H1	119.0	C26—C27—C28	119.8 (5)
C2—C1—H1	119.0	C26—C27—H27	120.1
C3—C2—C1	119.9 (5)	C28—C27—H27	120.1
C3—C2—H2	120.0	C27—C28—C29	117.1 (5)
C1—C2—H2	120.0	C27—C28—C30	125.6 (5)
C2—C3—C4	119.6 (5)	C29—C28—C30	117.3 (5)
C2—C3—H3	120.2	N5—C29—C28	122.9 (5)
C4—C3—H3	120.2	N5—C29—C33	116.3 (4)
C5—C4—C3	117.2 (5)	C28—C29—C33	120.8 (4)
C5—C4—C6	118.5 (5)	C31—C30—C28	122.1 (5)
C3—C4—C6	124.3 (5)	C31—C30—H30	118.9
N1—C5—C4	124.2 (5)	C28—C30—H30	118.9
N1—C5—C9	115.2 (4)	C30—C31—C32	121.3 (5)
C4—C5—C9	120.6 (5)	C30—C31—H31	119.3
C7—C6—C4	121.2 (5)	C32—C31—H31	119.3
C7—C6—H6	119.4	C34—C32—C33	117.5 (5)
C4—C6—H6	119.4	C34—C32—C31	124.7 (5)
C6—C7—C8	121.7 (5)	C33—C32—C31	117.8 (5)
C6—C7—H7	119.2	N6—C33—C32	123.3 (5)
C8—C7—H7	119.2	N6—C33—C29	116.0 (4)
C9—C8—C10	117.5 (5)	C32—C33—C29	120.6 (5)
C9—C8—C7	117.5 (5)	C35—C34—C32	119.1 (5)
C10—C8—C7	124.9 (5)	C35—C34—H34	120.5
N2—C9—C8	123.4 (5)	C32—C34—H34	120.5
N2—C9—C5	116.2 (4)	C34—C35—C36	120.7 (5)
C8—C9—C5	120.4 (5)	C34—C35—H35	119.7
C11—C10—C8	119.3 (5)	C36—C35—H35	119.7
C11—C10—H10	120.4	N6—C36—C35	122.0 (5)
C8—C10—H10	120.4	N6—C36—H36	119.0
C10—C11—C12	119.7 (5)	C35—C36—H36	119.0

### Hydrogen-bond geometry (Å, °)

**Table:**

*D*—H···*A*	*D*—H	H···*A*	*D*···*A*	*D*—H···*A*
C1—H1···Cl6^i^	0.93	2.80	3.416 (7)	125.
C11—H11···Cl2	0.93	2.82	3.740 (7)	172.
C12—H12···N4	0.93	2.55	3.038 (7)	113.
C25—H25···N3	0.93	2.62	3.098 (7)	113.
C36—H36···N2	0.93	2.60	3.084 (8)	113.

Symmetry codes: (i) *x*, *y*−1, *z*.
